# Reconstruction of a Rare Split‐Level Bilaminar Patellar Tendon Rupture With Concomitant Multiligament Knee Injury Using Peroneus Longus Autograft: A Case Report

**DOI:** 10.1155/cro/5640221

**Published:** 2026-09-30

**Authors:** Hamit Çağlayan Kahraman, Bekir Eray Kilinc

**Affiliations:** ^1^ Department of Orthopedics and Traumatology Fatih Sultan Mehmet Training and Research Hospital Istanbul Türkiye fsmhastanesi.gov.tr

**Keywords:** multiligamentous knee injury, patellar tendon rupture, peroneus longus autograft, split-level bilaminar rupture, surgical augmentation

## Abstract

**Introduction:**

Patellar tendon ruptures (PTRs) are critical causes of knee extensor mechanism failure. Although typically isolated, a split‐level bilaminar rupture pattern, in which different layers of the PTR at different anatomical levels, is extremely rare in adults, particularly when associated with complex multiligamentous injuries (occurring in only 0.02%–0.2% of orthopedic cases).

**Case Presentation:**

A 60‐year‐old female presented with acute knee pain and loss of active extension following a fall. Clinical examination revealed patella alta and a palpable infrapatellar gap. Imaging confirmed a full‐thickness PTR, a medial femoral condyle avulsion fracture (MCL attachment), and a fibular head fracture (PLC injury‐variant). Intraoperatively, a split‐level bilaminar PTR and a complete midsubstance ACL (anterior cruciate ligament) rupture were identified. The injury was managed through primary repair augmented with a peroneus longus tendon (PLT) autograft to provide early mechanical stability and protect secondary stabilizers during healing.

**Discussion:**

A split‐level bilaminar PTR in adults with concomitant multiligamentous injury is a rare clinical entity that requires high diagnostic suspicion. In this case, PLT augmentation provided a robust construct for extensor mechanism reconstruction while preserving the hamstring tendons and allowing good functional outcomes.

**Conclusion:**

In this case, anatomical repair augmented with a peroneus longus autograft resulted in restoration of the extensor mechanism at the 6‐month postoperative follow‐up while preserving the hamstring tendons.

**Highlights**

1. Rare injury pattern: describes a split‐level bilaminar patellar tendon rupture (PTR) associated with anterior cruciate ligament (ACL), medial collateral ligament (MCL), and posterolateral corner‐variant injuries in an adult patient.
2. Strategic graft selection: utilizes a peroneus longus tendon (PLT) autograft for augmentation to preserve hamstring tendons and protect secondary medial and rotatory knee stability.
3. Clinical outcome: primary repair combined with selective nonoperative management of the associated ligament injuries resulted in restoration of the extensor mechanism and satisfactory function in activities of daily living at 6 months.

## 1. Introduction

PTRs are critical causes of knee extensor dysfunction, leading to significant functional impairment [1]. Although typically resulting from high‐energy trauma or degenerative conditions, PTR combined with multiligament injuries (ACL, MCL, and fibular head fracture) is clinically rare, occurring in only 0.02%–0.2% of all orthopedic injuries [2, 3].

Split‐level bilaminar PTR, characterized by disruption of different tendon layers at distinct anatomical levels, is exceptionally rare in adults [4, 5]. Literature regarding the management of such complex cases with concomitant ligamentous instability is limited. This article describes a unique case of a split‐level bilaminar PTR associated with multiligament injuries managed with a primary repair augmented by a PLT autograft to ensure early stability while protecting secondary medial stabilizers.

## 2. Case Report

A 60‐year‐old female presented to the emergency department with severe left knee pain and an inability to bear weight after falling from a step. Physical examination revealed significant joint swelling, a palpable infrapatellar gap, and a high‐riding patella (patella alta). Active knee extension was absent. Distal pulses and neurological status were intact.

Radiographs showed a superiorly displaced patella and a small avulsion fragment at the medial femoral condyle (Figure 1). Computed tomography (CT) confirmed an avulsion fracture of the medial femoral condyle (MCL attachment) and a fracture of the fibular head (posterolateral corner injury). Magnetic resonance imaging (MRI) demonstrated a full‐thickness PTR with a high‐signal hematoma along the tendon sheath (Figure 2). Preoperative analysis of the MRI suggested a complex rupture, though the specific split‐level bilaminar nature was fully appreciated only intraoperatively.

**Figure 1:**
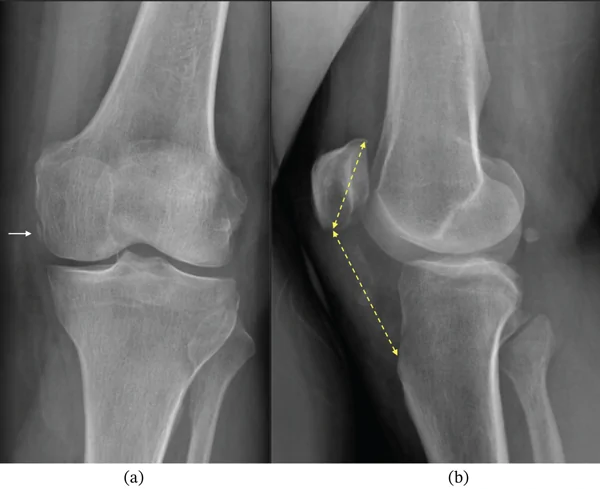
Preoperative radiographic evaluation of the left knee. (a) Anteroposterior view demonstrating a small avulsion fragment at the medial femoral condyle (white arrow). (b) Lateral view showing significant patella alta (Insall–Salvati ratio > 1.2) without evidence of joint dislocation.

**Figure 2:**
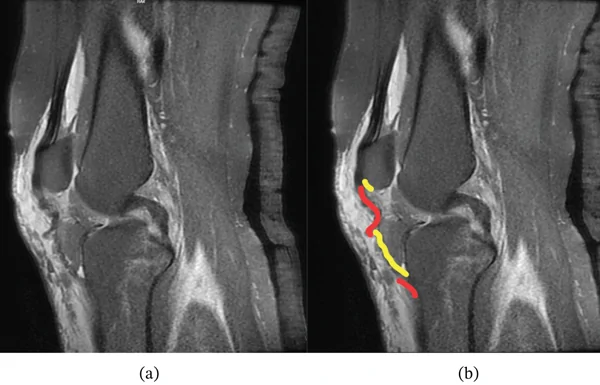
Preoperative advanced imaging demonstrating the complex rupture pattern. (a) Sagittal MRI showing the complete loss of tendon continuity and superior patellar tilt. (b) The red line indicates the superficial anterior fibers of the patellar tendon, whereas the yellow lines highlight the deep fibers in close proximity to the joint space.

The patient underwent urgent surgery. Under spinal anesthesia and tourniquet control, a longitudinal midline incision was performed. Intraoperative exploration revealed a unique laminar rupture pattern: The superficial (anterior) fibers were avulsed distally from the tibial tubercle, whereas the deep (posterior) fibers were avulsed from the inferior pole of the patella. This confirmed a split‐level bilaminar, full‐thickness PTR. Additionally, complete ACL midsubstance rupture was noted.

The surgical intervention began with the management of the deep laminar fibers, which were avulsed from the inferior pole of the patella. Two 2.5‐mm vertical transosseous tunnels were drilled through the patella from the distal edge to the proximal pole. The deep fibers were sutured using a Krakow locking technique with No. 2 FiberWire (Arthrex, Naples, Florida, United States). The suture ends were passed through the vertical tunnels and tied securely over the proximal patellar cortex. Subsequently, the superficial fibers—which had avulsed from the tibial tuberosity—were anatomically reduced and sutured onto the previously repaired deep layer near the tuberosity to restore the primary continuity of the tendon. Given the split‐level complexity of the rupture and the need for a robust construct permitting early mobilization, augmentation was deemed appropriate. To preserve secondary medial stabilizers and avoid further destabilization of the knee in the setting of the concomitant MCL injury, the hamstring tendons were strictly preserved. Instead, an ipsilateral PLT autograft (approximately 25 cm in length) was harvested. Transverse 4.5‐mm tunnels were created through the midpoint of the patella and the tibial tuberosity (Figure 3). The PLT graft was passed through the patellar and tibial tunnels. With the knee held in 30° of flexion, the graft was appropriately tensioned, and its ends were overlapped and sutured to one another. To reinforce the construct and protect the soft‐tissue fixation, a metallic U‐staple was used to secure the graft to the tibial tunnel at the medial aspect of the tuberosity (Figure 4). Finally, the medial and lateral retinacular tears were meticulously repaired using interrupted nonabsorbable sutures to complete the extensor mechanism reconstruction.

**Figure 3:**
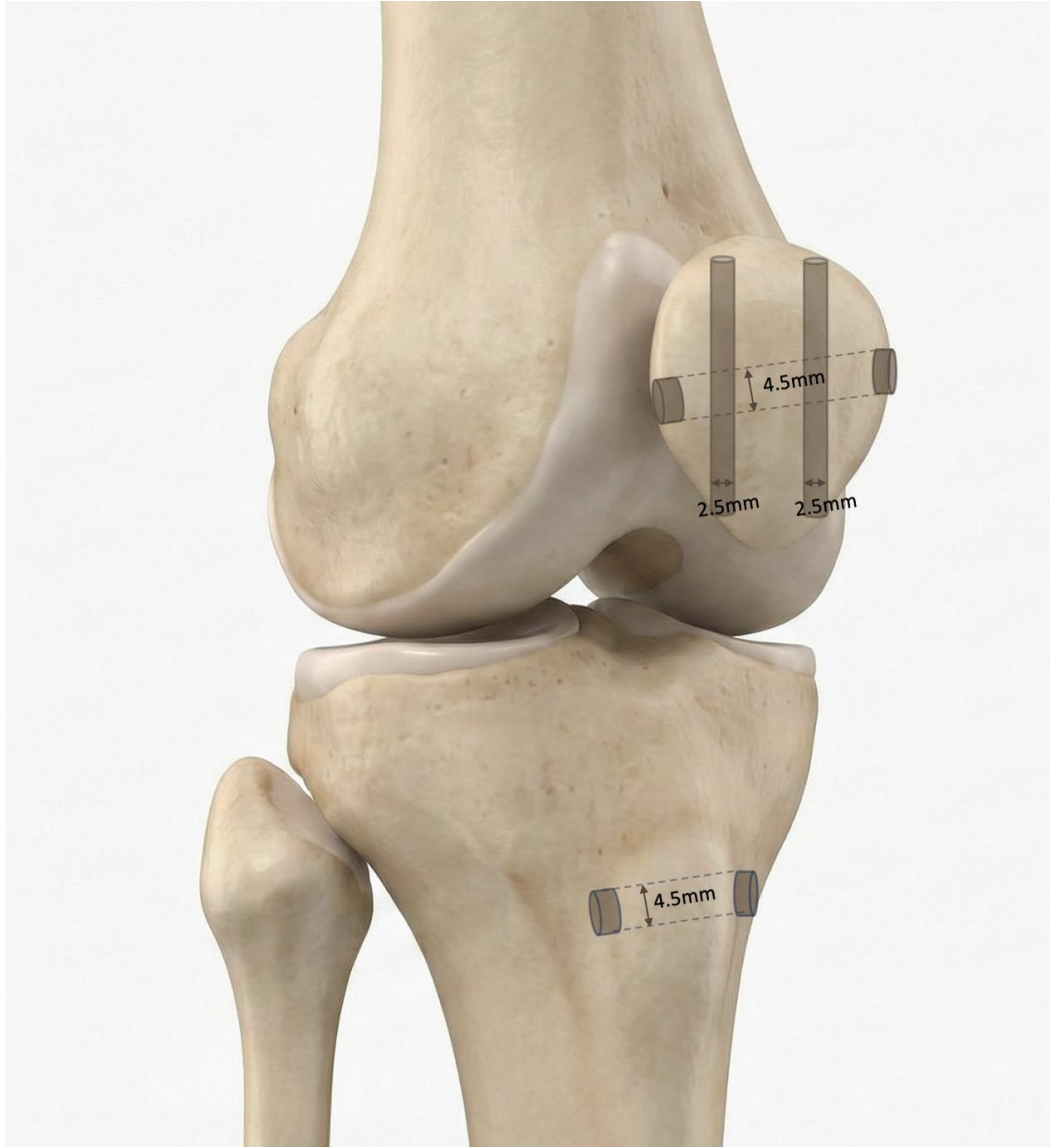
Schematic illustration of the surgical construct. Bone tunnel locations are highlighted: two vertical tunnels (2.5 mm) for the transosseous Krakow sutures of the deep laminar layer and two transverse tunnels (4.5 mm) in the midpatella and tibial tuberosity for the peroneus longus tendon autograft. The final configuration ensures a robust, dual‐level reinforcement of the extensor mechanism.

**Figure 4:**
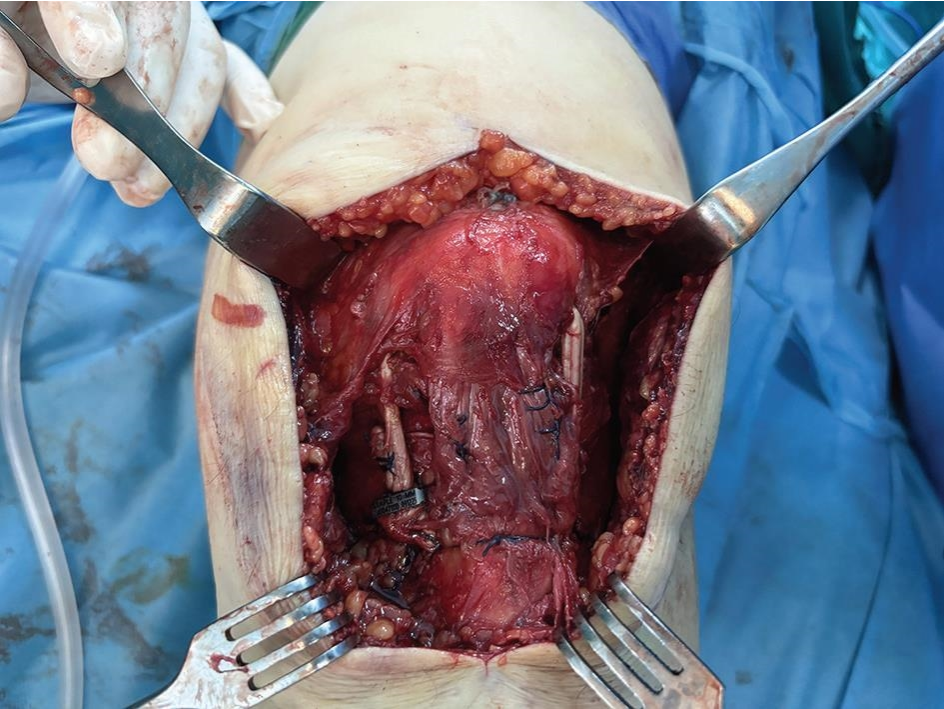
Intraoperative view of the repair. The deep fibers are secured at the proximal pole of the patella, whereas the superficial fibers are sutured at the distal anterior aspect of the patellar tendon. The peroneus longus tendon autograft is placed in an O‐shaped configuration and fixed with a metallic U‐staple.

Treatment of the associated ligament injuries was individualized according to functional demand and clinical necessity rather than chronological age alone. Prior to injury, the patient was independently ambulatory but did not participate in sports or other activities requiring high‐demand rotational stability; therefore, the primary treatment goal was restoration of pain‐free, independent ambulation and activities of daily living. Given the need for immediate extensor mechanism reconstruction and the additional morbidity and rehabilitation burden of concomitant multiligament reconstruction, ACL and PLC reconstructions were deferred, with staged reconstruction reserved for persistent symptomatic instability. The MCL injury was likewise managed conservatively, considering its recognized healing potential in selected multiligament knee injuries. In addition, the fibular head avulsion achieved solid union uneventfully during follow‐up. As the patient subsequently achieved satisfactory daily function without symptomatic instability, secondary ligament reconstruction was not required. This selective, function‐oriented strategy allowed adequate stability while avoiding unnecessary surgical morbidity (Table 1).

**Table 1:** Associated injuries identified in the present case and their initial management.

Injured structure	Specific lesion	Surgical management
Patellar tendon	Bilaminar (dual‐level) full‐thickness rupture	Primary transosseous repair (Krakow sutures) augmented with ipsilateral PLT autograft
MCL	Medial femoral condyle avulsion (Stieda fracture)	Conservative management (splinting)
ACL	Complete midsubstance rupture/tibial avulsion	Debridement; no reconstruction in the primary stage
Fibular head	Avulsion fracture (PLC injury variant)	Conservative management

Abbreviations: PLC, posterolateral corner; PLT, peroneus longus tendon.

Postoperative radiographic evaluation was performed (Figure 5), and the knee was immobilized in a long leg extension splint for 3 days. Deep vein thrombosis prophylaxis consisted of oral acetylsalicylic acid (Ecopirin Pro 81 mg) once daily for 30 days, together with the use of antiembolism stockings and early mobilization. Subsequently, early knee rehabilitation was initiated using a hinged knee brace, and assisted mobilization with the support of two crutches was commenced. The range of motion (ROM) was progressively increased on a weekly basis within the brace, permitting 30°, 60°, 90°, and 120° of flexion, respectively. By the second postoperative month, the patient was successfully mobilized without supportive aids, achieving a stable 120° of knee flexion. At the 6‐month postoperative follow‐up, the patient demonstrated complete restoration of the extensor mechanism without extension lag. Clinical stability assessment revealed a Grade 1/3 positive Lachman test, a Grade 1/3 positive anterior drawer test, and a Grade 0/3 pivot‐shift test. Varus stress testing at both 0° and 30° of knee flexion demonstrated only minimal side‐to‐side laxity compared with the contralateral knee. The Dial test showed no significant side‐to‐side difference at either 30° or 90° of knee flexion. The patient reported no episodes of giving‐way or subjective instability during activities of daily living. However, postoperative stress radiographs were not available.

**Figure 5:**
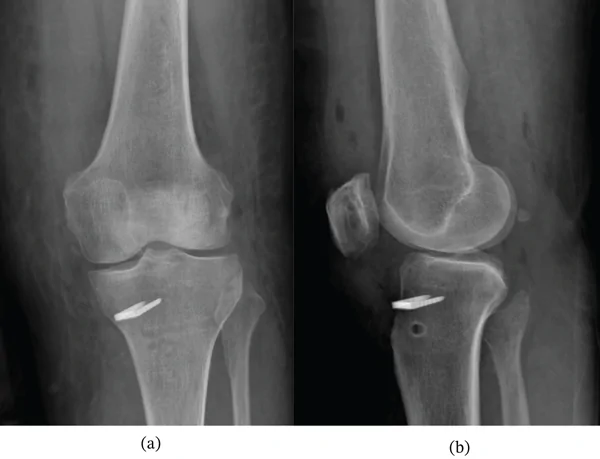
Postoperative radiographic evaluation. (a) Anteroposterior and (b) lateral views demonstrating the position of the transosseous bone tunnels in the patella and tibial tuberosity, as well as the stable placement of the metallic U‐staple used for the fixation of the peroneus longus tendon autograft.

At the 6‐month postoperative follow‐up, the patient showed satisfactory functional recovery. The Lysholm Knee Scoring Scale and Knee Injury and Osteoarthritis Outcome Score (KOOS) were self‐administered by the patient and reviewed by a member of the treating surgical team. The Lysholm knee score was 85, corresponding to a good clinical outcome, whereas the overall KOOS was 67%. KOOS subscale scores were 75% for symptoms/stiffness, pain, activities of daily living, and knee‐related quality of life, whereas the sport and recreational activities subscale score was lower at 35%. Additionally, the patient reported no pain, subjective instability, or functional limitation related to the donor ankle, and clinical examination revealed preserved ankle ROM without an apparent functional deficit. However, no validated ankle‐specific outcome measure was obtained.

## 3. Discussion

Simultaneous rupture of the patellar tendon and multiple knee ligaments is a devastating injury pattern, often categorized as a variant of knee dislocation. Although PTRs typically occur as single‐level avulsions—most commonly from the inferior pole of the patella—the bifocal pattern is exceedingly rare in the adult population [4]. Our case is characterized by an unusual split‐level bilaminar PTR in which the superficial fibers were avulsed distally from the tibial tubercle, whereas the deep fibers were avulsed proximally from the inferior pole of the patella. This pattern should be distinguished from previously reported bifocal PTRs, which involve disruption at two separate anatomical levels. Although bifocal patellar tendon injuries and PTRs associated with multiligament knee injuries have been described previously, the combination of a split‐level bilaminar rupture with concomitant ACL, MCL, and posterolateral corner injuries represents an exceptionally uncommon injury pattern.

A critical diagnostic pearl in this case was the discrepancy between preoperative imaging and intraoperative findings. Although sagittal MRI demonstrated a full‐thickness rupture with extensive high‐signal fluid tracking along the tendon, the specific laminar split was only fully appreciated during surgical exploration. The identified pattern—superficial fibers avulsed distally from the tibia and deep fibers avulsed proximally from the patella—creates a “split‐level” injury that challenges standard fixation techniques. This highlights that in complex extensor mechanism injuries where MRI demonstrates diffuse edema along the length of the patellar tendon, surgeons should maintain a high index of suspicion for a split‐level bilaminar failure. Although higher resolution MRI may theoretically facilitate visualization of complex tendon injuries, there is insufficient evidence to suggest that higher field strength alone would have identified this rare split‐level bilaminar pattern preoperatively [6, 7]. Careful evaluation of thin‐section multiplane images, particularly axial and sagittal sequences, could provide better differentiation of the various tendon layers.

PTRs concomitant with multiligament knee injuries are exceedingly rare and typically reported in younger populations following high‐energy trauma [8–10]. In contrast, our case involves an atypical injury pattern involving multiligamentous instability and a PTR in a 60‐year‐old patient following a low‐energy mechanism. Although bifocal PTRs involving injuries at two distinct anatomical levels have previously been reported, the present case differs in that the superficial and deep tendon layers failed at different levels, resulting in a split‐level bilaminar configuration [4, 11].

The choice of reconstruction graft was the most significant clinical decision in this case. Although hamstring tendons are frequently used for PT augmentation, they act as critical secondary stabilizers for medial and rotatory stability. In the presence of a concomitant Stieda‐type MCL avulsion and ACL tear, harvesting the hamstrings could have potentially compromised the residual stability of the knee. Therefore, we utilized an ipsilateral PLT autograft. Recent studies have demonstrated that PLT provides superior tensile strength—reported to be up to 1.8 times stronger than the semitendinosus—and offers sufficient length (typically > 20 cm) for augmentation without donor‐site morbidity at the knee joint [12]. The utilization of a PLT autograft for patellar tendon reconstruction has been previously documented as an effective salvage procedure, particularly in the complex setting of revision total knee arthroplasty [13]. In this case, we elected to apply this technique to our elderly patient, prioritizing the biomechanical advantages of the peroneal tendon to facilitate the most robust and expedited restoration of the extensor mechanism. This choice was specifically driven by the need for a high‐strength construct for atypical PTR that could withstand early mobilization and ensure reliable functional recovery in an older individual with limited biological healing potential.

The decision not to perform ACL and PLC reconstruction during the initial procedure was individualized and not based solely on the patient′s age. In the treatment decision‐making process following ACL injury, activity demands may be more influential than patient‐reported instability. In patients without high activity demands, a period of nonoperative management exceeding 3 months may help identify those who ultimately require surgical reconstruction and those who can be successfully managed conservatively [14]. In the present case, the patient′s preinjury functional demands were relatively low, prioritizing the restoration of independent walking and daily living activities. Furthermore, since the immediate surgical priority was the reconstruction of the complex split‐level bilaminar extensor mechanism injury, we chose to avoid additional ligament reconstruction during the same procedure and to reassess the need for staged reconstruction based on the residual symptomatic instability. During follow‐up, satisfactory functional improvement was achieved without clinically significant relevant symptomatic instability, and solid osseous union was achieved in the fibula head avulsion. Therefore, secondary ACL or PLC reconstruction was not undertaken.

The KOOS sport and recreational activities subscale score remained relatively low at 35%, which is an expected finding in an elderly patient following multiligamentous knee trauma, particularly when preinjury activity demands were limited. In this context, the primary therapeutic objective was restoration of pain‐free independent ambulation and activities of daily living rather than return to high‐demand rotational or athletic activities; therefore, the lower sport‐related score was not considered indicative of clinically relevant treatment failure. Although no clinically apparent donor‐site deficit was observed, the absence of a validated ankle‐specific outcome measure limits definitive conclusions regarding donor‐site morbidity.

This report has several limitations. First, it represents a single case, limiting the generalizability of the findings. Second, postoperative follow‐up is limited to 6 months, and longer term evaluation is needed to determine the sustainability of the clinical outcome. Finally, the treatment strategy and functional outcomes observed in this 60‐year‐old patient with relatively low functional demands should not be generalized to younger or more active patients with similar injury patterns.

In conclusion, split‐level bilaminar PTRs in adults are rare and in the setting of multiligament knee injuries require a meticulous surgical strategy that preserves secondary stabilizers. Restoration of the extensor mechanism and satisfactory function in activities of daily living were achieved, although residual ligamentous injury and limited sport‐related function should be considered when interpreting the overall outcome. The peroneus longus autograft may represent a useful alternative for patellar tendon augmentation when preservation of the hamstring tendons is desirable.

## Funding

No funding was received for this manuscript.

## Ethics Statement

This study is presented as a single case report; therefore, formal approval from the institutional ethics committee was not required according to local regulations. Written informed consent for publication of the clinical data and anonymized patient′s images was obtained from the patient.

## Consent

The patient provided written informed consent for the publication of her clinical information and anonymized images in this study.

## Conflicts of Interest

The authors declare no conflicts of interest.

## Data Availability

The datasets used and/or analyzed during the current study are available from the corresponding author on reasonable request.
